# Novel base catalysed rearrangement of sultone oximes to 1,2-benzisoxazole-3-methane sulfonate derivatives

**DOI:** 10.1186/1860-5397-3-20

**Published:** 2007-06-08

**Authors:** Veera Reddy Arava, Udaya Bhaskara Rao Siripalli, Vaishali Nadkarni, Rajendiran Chinnapillai

**Affiliations:** 1 Research and Development Laboratories, Suven Life Sciences Ltd., # 18, Phase – III, Jeedimetla, Hyderabad – 500055, India

## Abstract

A new process for the preparation of 1,2-benzisoxazole-3-methanesulfonates and 4-oximino-2,3-dihydrobenzoxathiin-2,2-dioxides (sultone oximes) is described. These compounds are important intermediates for the preparation of zonisamide, an anti-convulsant drug.

## Background

3-Alkyl-1,2-benzisoxazole derivatives are known to have important biological activities and are useful in different therapies. The 1,2-benzisoxazole moieties are isosteric with indoles and can mimic/bind to biologically important enzymes in a manner similar to indole derivatives. Out of many biologically active compounds, zonisamide is widely prescribed as an anti-epileptic drug. It was developed by Dianippon of Japan.

**Scheme 1:**
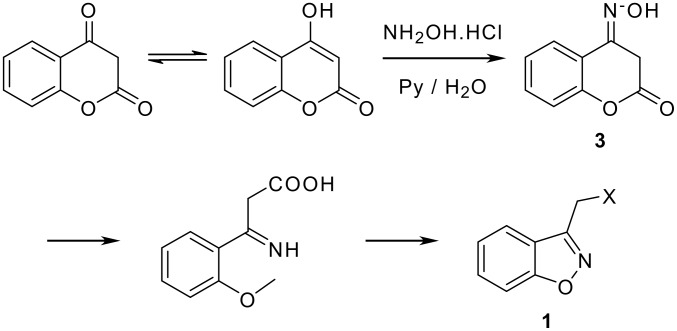
Conversion of coumarin-4-one oximes to 3-alkyl-1,2-benzisoxazole derivatives.

Compounds **1** were originally prepared from the rearrangement of coumarin-4-one oximes **3** as shown in Scheme 1.

**Scheme 2:**
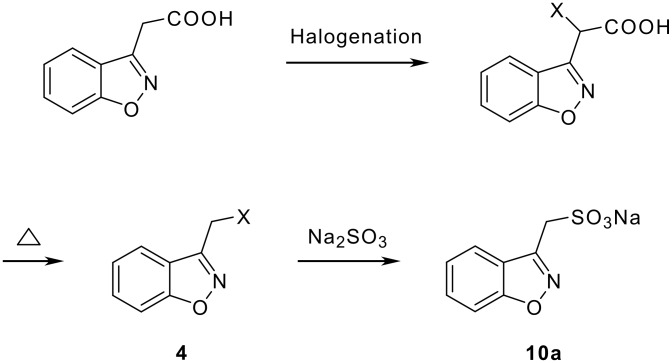
Reactions of 3-halomethyl derivatives with other nucleophiles.

The 1,2-benzisoxazole-3-acetic acids were converted into halogenated compounds, then reacted with various different nucleophiles to generate the compounds **1** as shown in Scheme 2.

Although the reactions in Scheme 2 were successfully implemented on large scale (~100 kgs), the lachrymatric nature of compound **4** (X = Br) created significant handling problems to the operating personnel. In our efforts to prepare the benzisoxazoles **10** without involving halogenated intermediates, we considered the replacement of the lactone carbonyl group in **3** with the SO_2_ group and studied the feasibility of the rearrangement of **5** to directly generate **10a**.

**Scheme 3:**
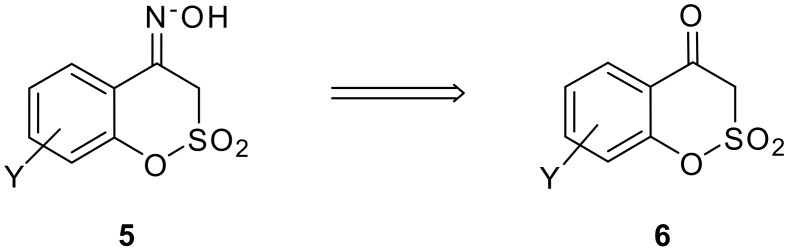
Sultone oximes and its precursor ketones.

**Scheme 4:**
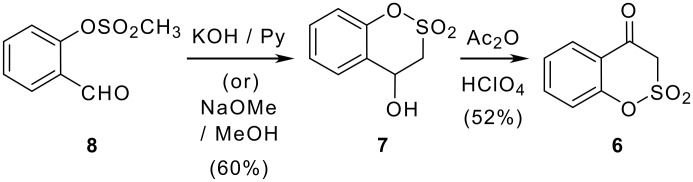
Synthesis of 1,2-benzoxathiin-4(3*H*)-one-2,2-dioxide **6** from methanesulfonate of salicylaldehyde.

The synthesis of compounds **5** has not been reported in the literature. Synthesis of compound **6** (1,2-benzoxathiin-4(3*H*)-one-2,2-dioxide Y = H), an obvious precursor for **5**, was reported by Timoney et al., in an overall yield of ~30% [1] via the cyclisation of the methanesulfonate of salicylaldehyde followed by oxidation (Scheme 4).

## Results and discussion

In order to prepare **6** in better yield, we choose methyl salicylate as the starting material and reacted it with methanesulfonyl chloride to provide the methanesulfonate derivative **9**. After exploring different reaction conditions with NaOH and KOH as bases, it was found that NaH in DMSO was optimal for the cyclisation. Other strong bases also gave good yields of **6**. The generality of the methodology has been established for several derivatives (Table 1). It was found that the reaction was efficient when groups such as Br, Cl, OMe & Me are present on the aromatic ring. On the other hand, the presence of nitro groups on the aromatic ring (3, 5 or 3,5-dinitro) failed to produce the cyclised products and gave only salicylic acid derivatives. All the 1,2-benzoxathiin-4(3*H*)-one-2,2-dioxides **6** were characterised from their analytical and spectral data.

**Scheme 5:**
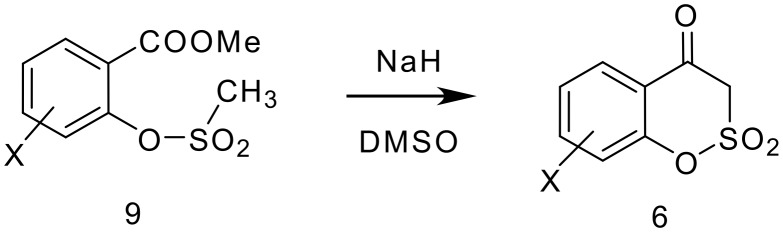
Preparation of 1,2-benzoxathiin-4(3*H*)-one-2,2-dioxides.

**Table 1:** Compounds **6a-i** prepared.

Compound	Substituent X in **9** **X**	Substituent X in **6** **X**	Yield (%)

**6a**	H	H	76
**6b**	5-Br	6-Br	58
**6c**	5-Cl	6-Cl	53
**6d**	3,5-dichloro	6,8-dichloro	47
**6e**	3,5-dibromo	6,8-dibromo	49
**6f**	3-OMe	8-OMe	62
**6g**	4-OMe	7-OMe	62
**6h**	5-OMe	8-OMe	63
**6i**	4-Me	7-Me	61

Conversion of ketones **6** into the corresponding oximes **5** proceeded uneventfully, and oximes **5** were characterised from the spectral data and physical properties (Table 2).

**Scheme 6:**
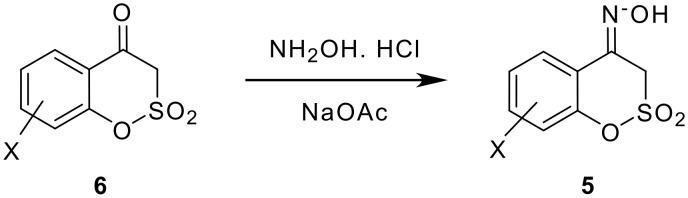
Preparation of sultone oximes.

**Table 2:** Compounds **5a-i** prepared.

Compound	Substituent X in **6** **X**	Yield (%)	M.P °C

**5a**	H	93	167–170
**5b**	6-Br	66	179–182
**5c**	6-Cl	62	138–140
**5d**	6,8-dichloro	58	178–180
**5e**	6,8-dibromo	58	180–182
**5f**	8-OMe	76	195–198
**5g**	7-OMe	76	160–162
**5h**	6-OMe	77	201–201
**5i**	7-Me	81	200–201

Although conversion of sultone oxime **5** into 3-alkyl-1,2-benzisoxazole derivatives [2–3] proved to be difficult using NaOH and KOH under different conditions, it was found that the rearrangement proceeded smoothly with either NaOH in ethylene glycol or with a solution of sodium methoxide in methanol. The yields are depicted in Table 3.

**Scheme 7:**
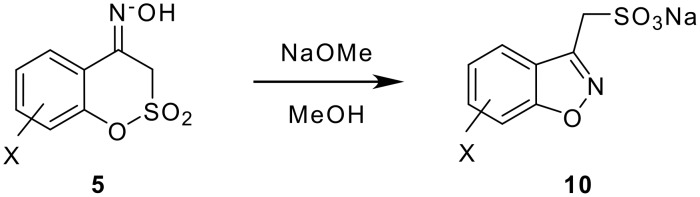
Preparation of 3-alkyl-1,2-benzisoxazole derivatives.

**Table 3:** Compounds **10a-i** prepared.

Compound	Substituent X in **5** X	Yield (%)	Substituent X in **10** X

**10a**	H	79	H
**10b**	6-Br	76	5-Br
**10c**	6-Cl	68	5-Cl
**10d**	6,8-dichloro	57	5,7-dichloro
**10e**	6,8-dibromo	59	5,7-dibromo
**10f**	8-OMe	72	7-OMe
**10g**	7-OMe	72	6-OMe
**10h**	6-OMe	69	5-OMe
**10i**	7-Me	75	6-Me

**Scheme 8:**
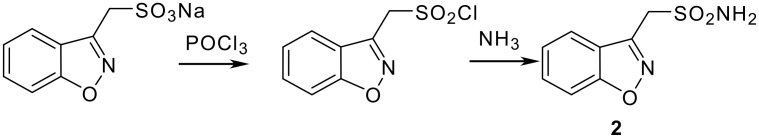
Synthesis of zonisamide from 1,2-benzisoxazole-3-methane-sulfonate.

The unsubstituted sodium 1,2-benzisoxazole-3-methane-sulfonate (**10a**) was converted into zonisamide employing the literature procedure (Scheme 8). [4]

## Conclusion

In summary, we have developed a simple and economical process for the preparation of 1,2-benzisoxazole-3-methanesulfonates from sultone oximes, which can be employed for the production of zonisamide and its derivatives. The 1,2-benzisoxazole-3-methanesulfonic acid sodium salt preparation has been filed as a PCT application. [5–6]

## Experimental

See Supporting Information File 1 for full experimental data.

## Supporting Information

File 1 The experimental section. The experimental data and the results of analysis.
