# Supplementary material for: Novel base catalysed rearrangement of sultone oximes to 1,2-benzisoxazole-3-methane sulfonate derivatives
Source: Beilstein J Org Chem. 2007 Jun 8;3:20. doi: 10.1186/1860-5397-3-20 (PMC1919382; doi:10.1186/1860-5397-3-20)
Supplement: File 1 — The experimental section. The experimental data and the results of analysis. [file Beilstein_J_Org_Chem-03-20-s001.doc]

**Experimental section**

Melting points were determined in open capillaries and are uncorrected. The purity of all the compounds was routinely checked by TLC on silica gel coated plates. IR spectra were recorded as KBr pellets using Perkin Elmer system 2000 FT IR spectrometer; ^1^H-NMR spectra were recorded on a Brucker 400 MHz instrument with TMS as internal standard (chemical shifts in δ, ppm) and mass spectra were recorded on a Perkin Elmer mass spectrometer operating at 70eV.

**Methyl 2-methanesulphonyloxybenzoate (9)**

To a stirred solution of methyl salicylate (100 g, 0.66 moles) in 300 mL of methylene chloride was charged triethylamine (80 g, 0.792 moles). The mixture was cooled to –5 to 0 °C and methanesulfonyl chloride (113 g, 0.99 moles) in methylene chloride (100 mL) was added at 0-5°C over a period of 1 h, maintained at 0°C for 1 h and raised to 22-25°C and maintained for 3 h. Water (200 mL) was added and stirred at 22-25 °C for 1 h. The organic layer was separated and the aqueous layer was extracted with 100 mL of methylene chloride. The combined organic layer was washed with 100 mL of water and the solvent was evaporated to give 160-162 g of methyl 2-methanesulphonyloxybenzoate (**9**), which was recrystallized from toluene to give 137 g of white solid (yield 90%).

M.p.: 48-51°C

GC Purity: > 99%

IR: ν _max_ (KBr) 1731, 1359, 1168 cm^-1^.

^1^H NMR (400 MHz, CDCl_3_): δ = 3.28 (s, 3H), 3.92 (s, 3H), 7.38 – 7.46 (m. 2H), 7.54 –

7.59 (m, 1H), 7.97 (dd, 1H, J_1_ = 2.4 Hz, J_2_ = 8.7 Hz).

^13^C NMR (100.6 MHz, CDCl_3_): δ = 38.4, 52.4, 124, 124.3, 127, 131.9, 133.6, 147.7,

164.5.

MS (m/z) = 231.1 (M+1).

Anal. Calcd for C_9_H_10_O_5_S: C, 46.95; H, 4.38; S, 13.93. Found: C, 46.72; H, 4.34; S, 13.6%.

**1,2-Benzoxathiin-4(3*H*)-one-2,2-dioxide (6a)**

A solution of dimethyl sulfoxide (250 mL) and sodium hydride (60%) (13 g, 0.326 moles) was heated to 70-80 °C under nitrogen and maintained at 75 °C for 4 h. The reaction mixture was cooled to 15 °C and methyl methanesulphonyloxybenzoate (**9a**; X = H) (52 g, 0.22 moles) in 50 mL of dimethyl sulfoxide was added over a period of 1 h. The temperature was raised to 20-25 °C and maintained for 2-3 h. Reaction mass was slowly quenched in to 2.5 L of water and was extracted with toluene (2 x 800 mL). The pH of the aqueous layer was adjusted to 5 with conc. HCl and extracted with ethyl acetate (2 x 750 mL). Evaporation of the solvent gave 34 g of 1,2-benzoxathiin-4(3*H*)-one-2,2-dioxide (**6a**) as light yellow solid (Yield: 76%).

Melting Point: 89-91°C

GC Purity: > 99%.

IR. ν _max_ (KBr): 1696, 1387, 1183 cm^-1^.

^1^H NMR (400 MHz, CDCl_3_): δ = 4.71 (s, 2H), 7.29-7.46 (m, 2H), 7.70-7.79 (dd, 1H, J_1_ =

2.5 Hz, J_2_ = 8.6 Hz), 8.13 (m, 1H)

^13^C NMR (100.6 MHz, CDCl_3_): δ = 59.6, 119.5, 120.4, 126.4, 128.8, 137.5, 153.8, 182.1.

MS (m/z) = 197 (M – 1).

Anal. Calcd for C_8_H_6_O_4_S: C, 48.48; H, 3.05; S, 16.18. Found: C, 48.32; H, 3.11; S, 15.96%.

**6-Bromo-1,2-benzoxathiin-4(3*H*)-one-2,2-dioxide (6b)**

M.p.: 128-129°C.

IR. ν_max_ (KBr): 1708, 1387, 1183 cm^-1^.

^1^H NMR (400 MHz, CDCl_3_): δ = 4.47 (s, 2H), 7.22 (d, 1H, J=8.0 Hz), 7.83(dd 1H, J_1_=2.5 Hz, J_2_=8.7 Hz), 8.2 (d, 1H, J=2.5 Hz).

^13^C NMR (100.6 MHz, CDCl_3_): δ = 59.6, 119.8, 121.5, 121.8, 131.5, 140.2, 152.8, 181.1.

Anal. Calcd for C_8_H_5_BrO_4_S: C, 34.67 ; H, 1.81; S, 11.57. Found: C, 34.49; H, 1.80; S, 11.56%.

**6-Chloro-1,2-benzoxathiin-4(3*H*)-one-2,2-dioxide (6c)**

M.p.: 122-125 °C.

IR. ν _max_ (KBr): 1699 cm^-1^.

^1^H NMR (400 MHz, CDCl_3_): δ = 4.47 (s, 2H), 7.27 (d, 1H, J=8.7 Hz), 7.68(dd 1H, J_1_=2.7 Hz, J_2_= 8.7 Hz), 8.05 (d, 1H, J=2.6 Hz).

^13^C NMR (100.6 MHz, CDCl_3_): δ = 59.5, 120.5, 121.8, 124.7, 129.9, 137.3, 152, 182.6.

Anal. Calcd for C_8_H_5_ClO_4_S: C, 41.3; H, 2.17; S, 3.78. Found: C, 41.24; H, 2.14; S, 13.76%.

**6,8-Dichloro-1,2-benzoxathiin-4(3*H*)-one-2,2-dioxide (6d)**

M.p.: 179-182 °C.

IR. ν_max_ (KBr): 1718 cm^-1^.

^1^H NMR (400 MHz, CDCl_3_): δ = 4.5 (s, 2H), 7.79 (d, 1H, J=2.5 Hz), 7.96 (d, 1H, J=2.6 Hz).

^13^C NMR (100.6 MHz, CDCl_3_): δ = 59.9, 120.7, 125.1, 133, 137.1, 148.5, 157.6, 182.2.

Anal. Calcd for C_8_H_4_Cl_2_O_4_S: C, 35.97; H, 1.50; S, 12.01. Found: C, 35.98; H, 1.53; S, 11.04%.

**6,8-Dibromo-1,2-benzoxathiin-4(3*H*)-one-2,2-dioxide (6e)**

M.p.: 168-173 °C.

IR. ν_max_ (KBr): 1713 cm^-1^.

^1^H NMR (400 MHz, CDCl_3_): δ = 4.5 (s, 2H), 8.08 (d, 1H, J=2.4 Hz), 8.15 (d, 1H, J=2.4 Hz).

^13^C NMR (100.6 MHz, CDCl_3_): δ = 59.4, 114.1, 119.1, 122.9, 129.9, 142.2, 149.6, 182.

Anal. Calcd for C_8_H_5_Br_2_O_4_S: C, 26.99; H, 1.13; S, 7.82. Found: C, 27.07; H, 1.16; S, 7.68%.

**8-Methoxy-1,2-benzoxathiin-4(3*H*)-one-2,2-dioxide (6f)**

M.p.: 135-138 °C.

IR. ν_max_ (KBr): 1703 (-C=O).

^1^H NMR (400 MHz, CDCl_3_): δ = 3.95 (s, 3H), 4.45 (s, 2H), 7.28 (dd, 1H, J_1_=2.0 Hz, J_2_=6.2 Hz, 7.33 (t, 1H, J=8.2 Hz), 7.65 (dd 1H, J_1_=1.9 Hz, J_2_=7.4 Hz).

^13^C NMR (100.6 MHz, CDCl_3_): δ = 56.5, 59.7, 119.3, 119.6, 121.4, 126.3, 143.6, 149.4, 182.5.

Anal. Calcd for C_9_H_8_O_5_S: C, 47.36; H, 3.94; S, 14.04. Found: C, 47.39; H, 3.98; S, 14.17%.

**7-Methoxy-1,2-benzoxathiin-4(3*H*)-one-2,2-dioxide (6g)**

M.p.: 115-116°C.

IR. ν_max_ (KBr): 1685 cm^-1^.

^1^H NMR (400 MHz, CDCl_3_): δ = 3.93 (s, 3H), 4.4 (s, 2H), 6.75(d,1H, J=2.4 Hz), 6.91(dd 1H, J_1_=2.4 Hz, J_2_=8.9 Hz), 8.02 (d,1H, J=8.8 Hz)

^13^C NMR (100.6 MHz, CDCl_3_): δ = 56.9, 59.6, 104.2, 113.9, 114.1, 130.2, 155.7, 166.9, 182.3.

Anal. Calcd for C_9_H_8_O_5_S: C, 47.36; H, 3.94; S, 14.04. Found: C, 47.28; H, 4.26; S, 14.22%.

**6-Methoxy-1,2-benzoxathiin-4(3*H*)-one-2,2-dioxide (6h)**

M.p.: 109-111 °C.

IR. ν_max_ (KBr): 1701 cm^-1^.

^1^H NMR (400 MHz, CDCl_3_): δ = 3.87 (s, 3H), 4.42 (s, 2H), 7.22 (d, 1H, J_1_=2.9 Hz, J_2_=7.6 Hz, 7.28 (dd, 1H, J_1_=2.9 Hz J_2_=9.1 Hz), 7.48 (d, 1H, J=2.9 Hz).

^13^C NMR (100.6 MHz, CDCl_3_): δ = 55.9, 59.4, 110.3, 120.9, 121.1, 124.6, 147.6 157, 183.6.

Anal. Calcd for C_9_H_8_O_5_S: C, 47.36 ; H, 3.94; S, 14.04. Found: C, 47.28; H, 3.93; S, 14.18%.

**7-Methyl-1,2-benzoxathiin-4(3*H*)-one-2,2-dioxide (6i)**

M.p.: 109-114 °C.

IR. ν_max_ (KBr): 1695 cm^-1^.

^1^H NMR (400 MHz, CDCl_3_) δ, 2.47 (s, 3H), 4.42 (s, 2H), 7.11 (d, 1H, J=2.4 Hz), 7.22 (dd 1H, J_1_=2.4 Hz, J_2_=8.1 Hz), 7.97 (d 1H, J=8.1 Hz).

^13^C NMR (100.6 MHz, CDCl_3_): δ = 21.3, 59.6, 118, 119.5, 127.5, 128, 149.8, 153.5, 183.2.

Anal. Calcd for C_9_H_8_O_5_S: C, 50.93; H, 3.79; S, 15.10. Found: C, 50.73; H, 3.93; S, 14.98%.

**4-Oximino-4,4-dihydro-1,2-benzoxathiin-2,2-dioxide (5a)**

To a stirred solution of hydroxylamine hydrochloride (17.6 g, 0.25 mole) aqueous sodium acetate (22 g, 0.27 moles in 100 mL water) and acetic acid (125 mL) was added **6a** (25 g, 0.123 moles) at rt. The reaction temperature was raised to 100 °C and maintained for 5 h. The reaction mixture was cooled to 20-25 °C and water (100 mL) was added under stirring. The reaction mixture was extracted with methylene chloride (3 x 500 mL) and the combined organic layer was evaporated to give crude oxime. The crude product was recrystallized from methanol to give 24 g of **5a** as white solid (Yield: 93%), HPLC Purity: > 99%.

M.p.: 167-170 °C.

IR. ν_max_ (KBr): 3276, 1372, 1168 cm^-1^.

^1^H NMR (400 MHz, DMSO-d_6_): δ = 5.06 (s, 2H), 7.28-7.37 (m, 2H), 7.48-7.56 (dd, 1H, J_1_ = 2.4 Hz, J_2_ = 8.7 Hz), 7.98 (m, 1H).

^13^C NMR (100.6 MHz, DMSO-d_6_): δ = 46.7, 118.9, 119.5, 124.4, 126.2, 131.8, 141.3, 150.2.

MS: (m/z) 214 (M+1).

Anal. Calcd for C_8_H_7_NO_4_S: C, 45.07; H, 3.31; N, 6.57; S, 15.04. Found: C, 44.82; H, 3.29; N, 6.55; S, 15.11%.

**6-Bromo-4-oximino-4,4-dihydro-1,2-benzoxathiin-2,2-dioxide (5b)**

M.p.: 179-182 °C.

IR. ν_max_ (KBr): 3461 cm^-1^.

^1^H NMR (400 MHz, DMSO-d_6_): δ = 5.09 (s, 2H), 7.33 (d, 1H, J=8.0 Hz), 7.7 (dd, 1H, J_1_=2.4 Hz, J_2_=8.7 Hz), 8.02 (d, 1H, J=2.4 Hz), 12.64 (s, 1H).

^13^C NMR (100.6 MHz, DMSO-d_6_): δ = 46.4, 118.3, 121, 121.7, 126.4, 134.3, 140.4, 149.2.

Anal. Calcd for C_8_H_6_BrNO_4_S: C, 32.89; H, 2.07; N, 4.79; S, 10.97. Found: C, 32.87; H, 2.17; N, 4.68; S, 10.83%.

**6-Chloro-4-oximino-4,4-dihydro-1,2-benzoxathiin-2,2-dioxide (5c)**

M.p.: 138-140 °C.

IR. ν_max_ (KBr): 3447 cm^-1^.

^1^H NMR (400 MHz, DMSO-d_6_): δ = 4.60 (s, 2H), 7.26 (d, 1H, J=8.7 Hz), 7.46 (dd, 1H, J_1_=2.5 Hz, J_2_=8.0 Hz), 7.9 (d, 1H, J=2.5 Hz).

^13^C NMR (100.6 MHz, DMSO-d_6_): δ = 46.4, 120, 121.5, 123.5, 130.5, 131.4, 140.6, 148.8.

Anal. Calcd for C_8_H_6_ClNO_4_S: C, 32.89; H, 2.07; N, 4.79; S, 10.97. Found: C, 32.61; H, 2.19; N, 4.94; S, 10.66%.

**6,8-Dichloro-4-oximino-4,4-dihydro-1,2-benzoxathiin-2,2-dioxide (5d)**

M.p.: 178-180 °C.

IR. ν_max_ (KBr): 3440 cm^-1^.

^1^H NMR (400 MHz, DMSO-d_6_): δ = 4.62 (s, 2H), 7.53 (d, 1H, J=2.4 Hz), 7.91 (d, 1H, J=2.5 Hz).

^13^C NMR (100.6 MHz, DMSO-d_6_): δ = 46.5, 122.2, 122.4, 124.5, 130.4, 131, 140.2, 144.7.

Anal. Calcd for C_8_H_5_Cl_2_NO_4_S: C, 34.06; H, 1.78; N, 4.96; S, 11.36. Found: C, 33.94; H, 1.80; N, 4.74; S, 11.21%.

**6,8-Dibromo-4-oximino-4,4-dihydro-1,2-benzoxathiin-2,2-dioxide (5e)**

M.p.: 180-182 °C.

IR. ν_max_ (KBr): 3462 cm^-1^.

^1^H NMR (400 MHz, DMSO-d_6_): δ = 4.5 (s, 2H), 7.66 (d, 1H, J=2.3 Hz), 8.02 (d, 1H, J=2.3 Hz), 12.2 (s, 1H).

^13^C NMR (100.6 MHz, DMSO-d_6_): δ = 46.5, 122.1, 122.5, 124.4, 130.1, 131, 140.6,

144.9.

Anal. Calcd for C_8_H_5_Br_2_NO_4_S: C, 25.90; H, 1.36; N, 3.78; S, 8.64. Found: C, 25.78; H,

1.34; N, 3.69; S, 8.62%.

**8-Methoxy-4-oximino-4,4-dihydro-1,2-benzoxathiin- 2,2-dioxide (5f)**

M.p.: 195-198 °C

IR. ν_max_ (KBr): 3283 cm^-1^.

^1^H NMR (400 MHz, DMSO-d_6_): δ = 3.91 (s, 3H), 4.6 (s, 2H), 7.02 (dd, 1H, J_1_=1.4 Hz, J_2_=8.2 Hz), 7.2 (t,1H, J=8.6 Hz), 7.55 (dd, 1H, J_1_=1.3 Hz, J_2_=8.2 Hz), 8.39 (s,1H).

^13^C NMR (100.6 MHz, DMSO-d_6_): δ = 46.5, 56.1, 114.2, 115.2, 119.9, 126, 139.6, 141.7, 149.

Anal. Calcd for C_9_H_9_NO_5_S: C, 44.44; H, 3.72; N, 5.75; S, 13.1. Found: C, 44.36; H, 3.98; N, 5.46; S, 12.70%.

**7-Methoxy-4-oximino-4,4-dihydro-1,2-benzoxathiin 2,2-dioxide (5g)**

M.p.: 160-162 °C.

IR. ν_max_ (KBr): 3282 cm^-1^.

^1^H NMR (400 MHz, DMSO-d_6_): δ = 3.86 (s, 3H), 4.5 (s, 2H), 6.67 (d, 1H, J=2.5 Hz), 6.84 (dd, 1H, J_1_=2.6 Hz, J_2_=8.9 Hz), 7.9 (d, 1H, J=8.9 Hz), 8.1 (s, 1H, -OH).

^13^C NMR (100.6 MHz, DMSO-d_6_): δ = 46.4, 55.9, 103.9, 111.3, 113.5, 125.5, 141, 151.2, 161.8.

Anal. Calcd for C_9_H_9_NO_5_S: C, 44.44; H, 3.72; N, 5.75; S, 13.1. Found: C, 44.39; H, 3.68; N, 5.68; S, 13.23%.

**6-Methoxy-4-oximino-4,4-dihydro-1,2-benzoxathiin-2,2-dioxide (5h)**

M.p.: 199-202 °C.

IR. ν_max_ (KBr): 3335 cm^-1^.

^1^H NMR (400 MHz, DMSO-d_6_): δ = 3.83 (s, 3H), 4.5 (s, 2H), 6.99 (dd, 1H, J_1_=3.1 Hz, J_2_=9.1 Hz), 7.11 (d, 1H, J=9.1 Hz), 7.52 (d, 1H, J=3.1 Hz), 7.92 (s, 1H, -OH).

^13^C NMR (100.6 MHz, DMSO-d_6_): δ = 46.4, 55.6, 107.4, 118.3, 119.7, 120.6, 141.5, 144.2, 156.7.

Anal. Calcd for C_9_H_9_NO_5_S: C, 44.44; H, 3.72; N, 5.75; S, 13.1. Found: C, 44.38; H, 3.65; N, 5.76; S, 13.63%.

**7-Methyl-4-oximino-4,4-dihydro-1,2-benzoxathiin-2,2-dioxide (5i)**

M.p.: 200-201 °C.

IR. ν_max_ (KBr): 3279 cm^-1^.

^1^H NMR (400 MHz, DMSO-d_6_): δ = 2.4 (s, 3H), 4.58(s, 2H), 6.99 (d, 1H, J=2.6 Hz), 7.09 (dd, 1H, J_1_=2.6 Hz, J_2_=8.1 Hz), 7.86 (d, 1H, J=8.2 Hz), 7.92 (s, 1H, -OH).

^13^C NMR (100.6 MHz, DMSO-d_6_): δ = 20.7, 46.6, 116.2, 119.4, 124, 127, 141.3, 142.5, 150.

Anal. Calcd for C_9_H_9_NO_4_S: C, 47.57; H, 3.99; N, 6.16; S, 14.11 Found: C, 47.38; H, 3.92; N, 6.08; S, 14.31%.

**Sodium 1,2-benzisoxazole-3-methanesulfonate (10a)**

To a stirred solution of 4-oximino-4,4-dihydro-1,2-benzoxathiin 2,2-dioxide (**5a**) (10 g, 0.047 moles) in 50 mL of methanol at rt was added 22% sodium methoxide in methanol solution (4.43 g in 20 mL of methanol, 0.082 mole) at 25-30 °C over a period of 30-40 min. The color of the solution changed to yellow and the solid started precipitated from the clear solution. The solution was stirred at rt for 10 h. Methanol was evaporated under vacuum. To the residue obtained, methanol was added (300 mL) and heated to 50-60 °C to get a clear solution. The clear solution was treated with 1 g of activated charcoal and filtered. The filtrate was concentrated to 65-70 mL volume and was cooled to 0-5 °C. The product was filtered, washed with chilled methanol to get the title compound **10a** as a white crystalline solid.

^1^H NMR (400 MHz, D_2_O): δ = 4.64 (s, 2H), 7.41-7.47 (m, 2H), 7.66-7.69 (m, 1H), 7.91 (dd, 1H, J_1_ = 2.0 Hz, J_2_ = 8.8 Hz).

^13^C NMR (100.6 MHz, D_2_O): δ = 47, 109.5, 120.3, 122.4, 124, 130.7, 151.8, 162.8.

Anal. Calcd for C_8_H_6_NNaO_4_S: C, 40.85; H, 2.57; N, 5.96; S, 13.63. Found: C, 40.52; H, 2.53; N, 5.91; S, 13.44%.

**Sodium 5-bromo-1,2-benzisoxazole-3-methanesulfonate (10b)**

^1^H NMR (400 MHz, D_2_O): δ = 4.16 (s, 2H), 7.67 (d, 1H, J=8.0 Hz), 7.74 (dd, 1H, J_1_=2.0 Hz, J_2_=8.8 Hz), 8.24 (d,1H, J=1.6 Hz).

^13^C NMR (100.6 MHz, D_2_O): δ = 47.7, 111.3, 115, 123.6, 126.9, 132.5, 153.5, 161.4.

Anal. Calcd for C_8_H_5_BrNNaO_4_S: C, 30.59; H, 1.60; N, 4.46; S, 10.21. Found: C, 30.51; H, 1.63; N, 4.38; S, 10.13%.

**Sodium 5-chloro-1,2-benzisoxazole-3-methanesulfonate (10c)**

^1^H NMR (400 MHz, D_2_O): δ = 4.17 (s, 2H), 7.61 (d, 1H, J=8.8 Hz), 7.73 (dd, 1H, J_1_=1.8 Hz, J_2_=8.8 Hz), 8.09 (d, 1H, J= 1.8 Hz).

^13^C NMR (100.6 MHz, D_2_O): δ = 47.7, 110.9, 123, 123.8, 127.3, 130, 153.6, 161.

Anal. Calcd for C_8_H_5_ClNNaO_4_S: C, 35.64; H, 1.87; N, 5.19; S, 11.89. Found: C, 35.48; H, 1.85; N, 5.21; S, 11.82%.

**Sodium 5,7-dichloro-1,2-benzisoxazole-3-methanesulfonate (10d)**

^1^H NMR (400 MHz, D_2_O): δ = 4.17 (s, 2H), 8.10 (d, 1H, J=2.1 Hz), 8.2 (d, 1H, J=2.1 Hz).

^13^C NMR (100.6 MHz, D_2_O): δ = 47.9, 111.8, 114, 123.8, 127.3, 132.1, 153.2, 161.5.

Anal. Calcd for C_8_H_4_Cl_2_NNaO_4_S: C, 31.60; H, 1.33; N, 4.61; S, 10.54. Found: C, 31.48; H, 1.29; N, 4.43; S, 10.52%.

**Sodium 5,7-dibromo-1,2-benzisoxazole-3-methanesulfonate (10e)**

^1^H NMR (400 MHz, D_2_O): δ = 4.19 (s, 2H), 8.23 (d, 1H, J=1.7 Hz), 8.6 (d, 1H, J=1.7 Hz).

^13^C NMR (100.6 MHz, D_2_O): δ = 47.6, 110.5, 118, 125.2, 128.4, 133.8, 154, 160.9.

Anal. Calcd for C_8_H_4_Br_2_NNaO_4_S: C, 24.45; H, 1.03; N, 3.56; S, 8.16. Found: C, 24.35; H, 1.08; N, 3.52; S, 8.14%.

**Sodium 7-methoxy-1,2-benzisoxazole-3-methanesulfonate (10f)**

^1^H NMR (400 MHz, D_2_O): δ = 3.95 (s, 3H), 4.12 (s, 2H), 7.13 (dd, 1H, J_1_=1.4 Hz, J_2_=7.3 Hz), 7.25 (t, 1H, J=7.8 Hz), 7.55 (dd, 1H, J_1_=1.4 Hz, J_2_=7.8 Hz).

^13^C NMR (100.6 MHz, D_2_O): δ = 47.8, 56.2, 110.9, 115.6, 123.3, 124.3, 143.6, 152.7, 153.9.

Anal. Calcd for C_9_H_8_NNaO_5_S: C, 40.76; H, 3.04; N, 5.28; S, 12.09. Found: C, 40.71; H, 2.95; N, 5.25; S, 12.12%.

**Sodium 6-methoxy-1,2-benzisoxazole-3-methanesulfonate (10g)**

^1^H NMR (400 MHz, D_2_O): δ = 3.83 (s, 3H), 4.07 (s, 2H), 6.92 (dd, 1H, J_1_=2.1 Hz, J_2_=8.7

Hz), 7.19 (d, 1H, J=2.0 Hz), 7.87 (d, 1H, J=8.7 Hz).

^13^C NMR (100.6 MHz, D_2_O): δ = 47.8, 55.9, 92.2, 113.5, 114.9, 124.7, 153.3, 161.7,

164.4.

Anal. Calcd for C_9_H_8_NNaO_5_S: C, 40.76; H, 3.04; N, 5.28; S, 12.09. Found: C, 40.58; H,

2.99; N, 5.21; S, 11.83%.

**Sodium 5-methoxy-1,2-benzisoxazole-3-methanesulfonate (10h)**

^1^H NMR (400 MHz, D_2_O): δ = 3.77 (s, 3H), 4.13 (s, 2H), 7.2 (dd, 1H, J_1_=2.6 Hz, J_2_=9.1 Hz), 7.48 (d, 1H, J=2.6 Hz), 7.57 (d, 1H, J=9.1 Hz).

^13^C NMR (100.6 MHz, D_2_O): δ = 47.8, 55.7, 104.8, 109.8, 119.8, 122.1, 153.7, 155.5, 157.9.

Anal. Calcd for C_9_H_8_NNaO_5_S: C, 40.76; H, 3.04; N, 5.28; S, 12.09. Found: C, 40.63; H, 3.11; N, 5.18; S, 12.02%.

**Sodium 6-methyl-1,2-benzisoxazole-3-methanesulfonate (10i)**

^1^H NMR (400 MHz, D_2_O): δ = 2.48 (s, 3H), 4.10 (s, 2H), 7.14 (dd, 1H, J_1_=1.6 Hz, J_2_=8.2 Hz), 7.45 (d, 1H, J=1.6 Hz), 7.89 (d, 1H, J=8.2 Hz).

^13^C NMR (100.6 MHz, D_2_O): δ = 21.4, 47.8, 108.7, 119.4, 123.8, 124.7, 140.3, 153.4, 163.

Anal. Calcd for C_9_H_8_NNaO_4_S: C, 43.37; H, 3.24; N, 5.62; S, 12.87. Found: C, 43.14; H, 3.16; N, 5.38; S, 12.65%.
